# A Flexible Laser-Induced Graphene Memristor with Volatile Switching for Neuromorphic Applications

**DOI:** 10.1021/acsami.4c07589

**Published:** 2024-09-06

**Authors:** Mohit D. Ganeriwala, Roberto Motos Espada, Enrique G. Marin, Juan Cuesta-Lopez, Mikel Garcia-Palomo, Noel Rodríguez, Francisco G. Ruiz, Andres Godoy

**Affiliations:** Electronics Department, Campus Fuentenueva S/N, University of Granada, Granada 18071, Spain

**Keywords:** laser-induced graphene, memristors, 2D materials, neuromorphic, synapse, LIF neuron

## Abstract

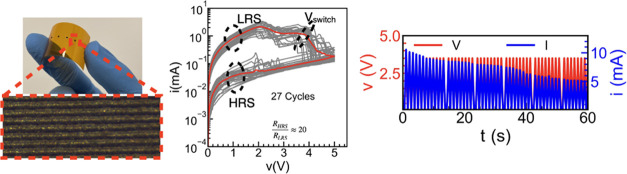

Two-dimensional graphene and graphene-based materials are attracting increasing interest in neuromorphic computing applications by the implementation of memristive architectures that enable the closest solid-state equivalent to biological synapses and neurons. However, the state-of-the-art fabrication methodology involves routine use of high-temperature processes and multistepped chemical synthesis, often on a rigid substrate constraining the experimental exploration in the field to high-tech facilities. Here, we demonstrate the use of a one-step process using a commercial laser to fabricate laser-induced graphene (LIG) memristors directly on a flexible polyimide substrate. For the first time, a volatile resistive switching phenomenon is reported in the LIG without using any additional materials. The absence of any precursor or patterning mask greatly simplifies the process while reducing the cost and providing greater controllability. The fabricated memristors show multilevel resistance-switching characteristics with high endurance and tunable timing characteristics. The recovery time and the trigger pulse-dependent state change are shown to be highly suitable for its use as a synaptic element and in the realization of leaky-integrate and fire neuron in neuromorphic circuits.

## Introduction

In recent years, there exist an increasing interest in promoting a novel computing paradigm able to mimic the complex behavior of biological brains.^1−3^ At the leading edge of this revolutionary change are Spiking Neural Networks (SNNs) which attempt to efficiently translate the biological neural functioning into an artificial hardware.^4,5^ The existing implementations of SNNs, however, usually involve a large number of active CMOS components preventing these architectures from the inherent high-density integration of the real biological networks.^6−8^ In order to overcome the CMOS intrinsic limitations, several emerging devices based on different physical mechanisms have been proposed, including phase change materials or magneto-electric-controlled ferromagnets, among others.^2^ However, these structures face severe limitations, such as stringent operating conditions and unsuitable switching properties, compromising the emulation of a realistic neural network. In this landscape, resistive switching memristors have emerged as a favorable choice for realizing SNNs with minimum footprint.^9,10^ In particular, two-terminal devices using a wide range of metallic electrodes sandwiching a bulk insulator have already demonstrated both nonvolatile and volatile switching behavior,^11,12^ emulating, respectively, the synapse retentive long-term plasticity (LTP)^13,14^ and dynamic short-term plasticity (STP).^10,15^

Recent studies have revealed that layered two-dimensional (2D) materials, such as graphene, transition metal dichalcogenides (TMDCs), and insulating 2D materials, are able to reproduce and enhance the functionality of memristive devices.^16,17^ A paradigmatic advantage of 2D materials is found with their deposition on flexible substrates,^18−20^ that could find a direct application on future neural interface technologies, enabling, e.g., to connect and interact with living neuronal networks or to recover the processing capabilities lost by neurodegeneration.^21^

The state-of-the-art fabrication methodology for 2D materials involves, nevertheless, routine use of high-temperature, time-consuming processes, and multistepped chemical synthesis, as exemplified by many of the graphene-^17,22−24^ and other 2D materials-based^25,26^ memristors reported in the literature, constraining the experimental exploration in this field to high-tech facilities. In this context, Laser-Induced Graphene (LIG) has emerged as a cost-effective alternative technique for the production and patterning of graphene films using commercial laser machines on flexible substrates.^27,28^ LIG has already shown high electrical conductivity (25 S·cm^–1^), and good thermal stability (>900 °C).^28,29^ Additionally, patterning- and lithography-free fabrication process is allowed on a flexible substrate, with a remarkable control of the porosity, defect density, and geometry by adjusting the laser type and parameters such as the applied power and speed.^28,30^ The unique features of LIG have already been employed to develop numerous applications in the area of flexible electronics and energy storage devices such as sensors and supercapacitors.^31,32^ The implementation of memristors on a flexible substrate both with LIG and laser-reduced graphene oxide (rGO) has also been reported.^33−36^ In particular, in the works by Tian et al.^33^ and Fatima et al.^34^ laser scribing was used to generate rGO from a precursor GO film. In both studies, rGO was used only as one of the electrodes, while the active resistive switching material was HfO_2_ and MXene, respectively. Interestingly, the laser-fabricated rGO in ref (33) has been shown to not exhibit resistive switching behavior on its own. On the other hand, the work by Romero et al.^36^ used a laser to reduce the GO film and it did demonstrate resistive switching directly on rGO. However, any work using laser rGO, as the aforementioned, requires additional preprocessing steps to prepare the precursor GO film, not needed in the LIG process. Another study by Enaganti et al.^35^ used laser-fabricated graphene by directly scribing a polymer film to demonstrate a memristor. The memristor reported in this later work showed similar nonvolatile bipolar resistive switching behavior as the ones with rGO. While, the nonvolatile memristors have a diverse set of applications such as memories, the volatile self-recovering memristors (as the one presented in our work) have gained special attention aiming at different specialized applications such as selectors for memristive crossbar arrays,^37^ artificial neurons in neuromorphic computing,^38,39^ and true random number generators (TRGN),^40^ based on their recovery time.^41^

In this work, we employ a one-step process exploiting a commercial laser to implement for the first time a Volatile-Laser-induced graphene Memristor (VLM) on a flexible polyimide substrate. The absence of any precursor and patterning mask enables a substantial simplification of the process, considerably reducing the manufacturing time and cost. The following is the organization of the paper: First, the structural properties of the VLM are described, followed by a discussion on the electrical performance. The physical mechanism governing the resistive switching is elucidated next by utilizing numerical device simulations and experimental measurements. Afterward, the potential of the fabricated devices to emulate some relevant features of biological synapses is shown, and finally, the main conclusions of the work are presented.

## Results and Discussion

### Structural Properties

**Figure 1:**
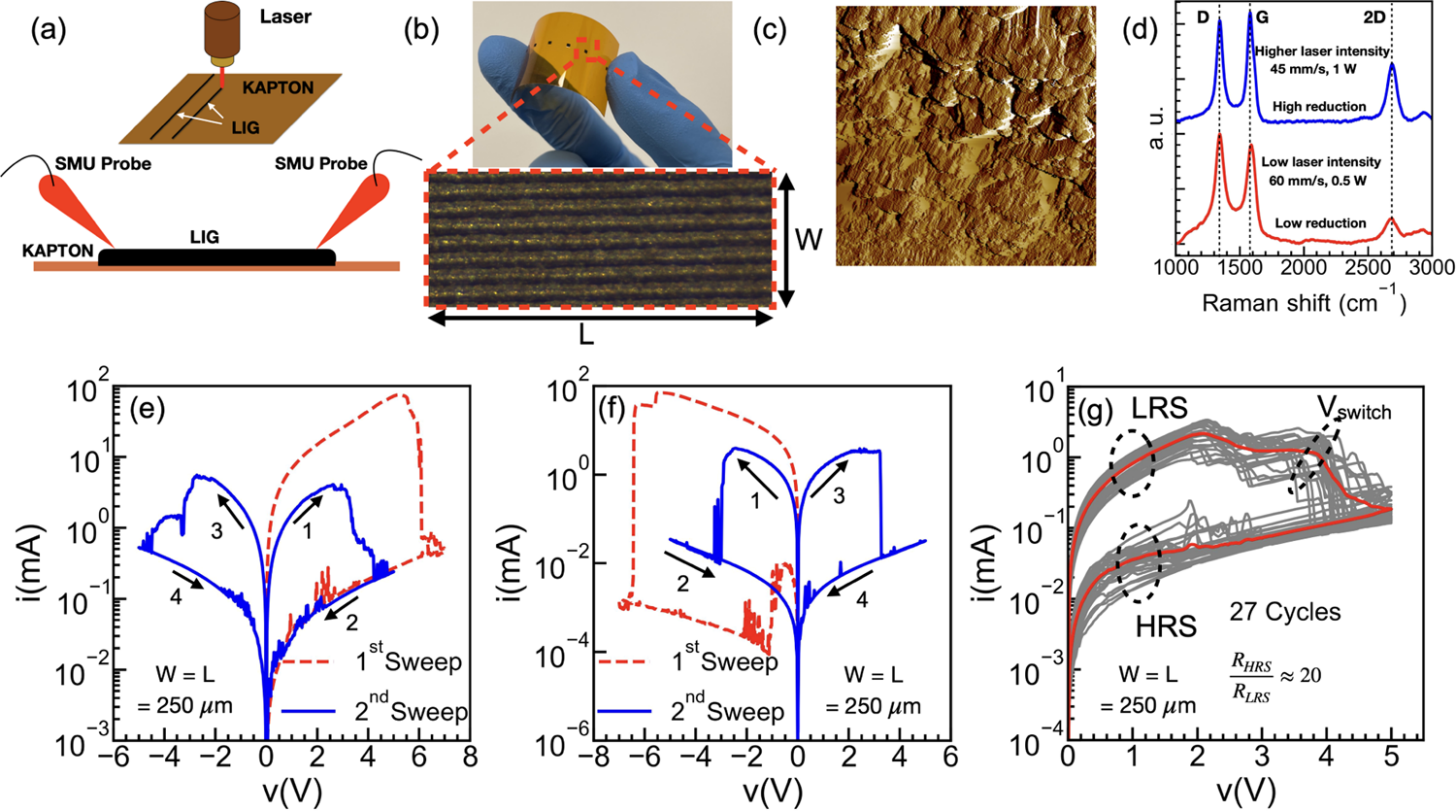
(a) Schematic representation of the laser engraving process resulting in closely packed lines on the polymer substrate and the use of SMU probes as contacts. (b) Image of a series of fabricated volatile LIG memristors (VLM). The zoomed image shows a close-up of one of the VLM with length *L* and width *W*. (c) Atomic force microscopy (AFM) image of the engraved surface. (d) Raman spectroscopy result of two VLMs obtained from two different laser intensity and speed settings. (e) Measured current–voltage (*I*–*V*) characteristics of the VLM, starting with positive polarity for the voltage sweep corresponding to the forming loop (red line) and subsequent working loop (blue line). (f) *I*–*V* characteristics of the VLM, starting with negative polarity for the voltage sweep of the forming loop (red line) and subsequent working loop (blue line). (g) Repeated *I*–*V* measurements for 27 cycles (gray); red line shows the median data.

As schematically depicted in Figure 1a, LIG is generated by laser engraving the polyimide film. The chemical structure of the employed polyimide, which is insulating in its pristine form, is a polymer chain of sp^3^ and sp^2^ bonded carbon atoms with C–O, C=O, and C–N bonds (see Figure S1). The laser delivers localized heating to the substrate, originating the breaking of the carbon–oxygen and carbon–nitrogen bonds and giving rise to a rearrangement of the residual carbon chains and release of the gaseous byproduct.^30^Figure 1b,1c, respectively, show close-up optical microscopy and Atomic Force Microscopy (AFM) images of the scribed region corresponding to the dark zone on the flexible polyimide substrate. Here, *L* and *W* stand for the length and width of the VLM, respectively. The laser scriber is arranged to engrave a series of closely packed lines on the substrate, as shown in the zoomed-in view of Figure 1b.

The Raman spectra of the resulting material are represented in Figure 1d for two different scenarios: first one corresponding to a high speed and low applied power (red line) and a second one (blue line) with lower speed and higher power. Both combinations depict the characteristic D, G, and 2D peaks.^28^ The G peak appears at 1590 cm^–1^ when the polymer is exposed to low laser intensity, and becomes narrower and shifts to 1580 cm^–1^ for higher power, signifying the re-establishment of the sp^2^ carbon bonding and the reduction in the oxygen content.^42^ The occurrence of a broader D peak at 1350 cm^–1^, associated with the presence of structural defects in graphene, is also reduced in intensity and width with increasing laser power.^43,44^ The most prominent feature of the graphene sheet, the 2D peak, is highly incremented with an increasing laser power, and its appearance at 2700 cm^–1^ indicates the presence of few layers of graphene.^42^ According to the selected laser power and its engraving speed, it is therefore possible to tune the material properties and, in principle, the electrical conductivity of the exposed region.

### Electrical Performance

The measured current–voltage (*I*–*V*) characteristics of two VLMs with square cross sections *W* = *L* = 250 μm are shown in Figure 1e,f. First, a triangular voltage sweep with different polarity is applied to the pristine sample, initially positive (from 0 to 6 V and back) for Figure 1e and negative (from 0 to −6 V and back) for Figure 1f, respectively. The measured current (dashed red line) shows hysteresis, with its value switching between two clearly distinguishable resistance states. This first hysteresis loop is characterized by a switching voltage (*V*_switch_) of ≈5.2 and −6.2 V, with a maximum current value of around 74 and 69 mA, respectively, in Figure 1e,1f. Note that *V*_switch_ is defined here as the voltage where the current decreases by 10% of the maximum value. After the initial I–V loop, which resembles the forming process usually observed in metal oxide memristors,^12^ subsequent triangular biasing signals (with amplitude ranging from 5 to −5 V) are applied. The resulting *I*–*V* cycles (termed as the working loops here and plotted as solid blue lines in Figure 1e,1f) present reduced *V*_switch_ and current with a rather symmetric response. In more detail, *V*_switch_ ∼± 3 and ∼±2.5 V and maximum current values of 5 and 4 mA are observed in the working loops of Figure 1e,1f, respectively. The off-current of the device, *I*_off_, measured at *V*_read_ = 0.1 V, is in the range of 7–0.1 μA, which provides an *I*_max_/*I*_off_ ratio around 10^3^. These high operating currents due to the wide cross-sectional area could, in principle, be reduced by improving the laser resolution.

Notably, the direction of the hysteresis of the VLMs, depicted by the numbered arrows in Figure 1e,1f, is reversed (i.e., clockwise) as compared to the usual volatile memristors.^25^ The VLMs start in a low resistance state (LRS) and switch to the high resistance state (HRS) at *V*_switch_. After a recovery time on the removal of the bias, the VLM returns to the LRS, showing volatile and diffusive ionic characteristics. The switching behavior is similar for both voltage polarities and is also independent of the initial forming voltage polarity. Figure 1g shows 27 *I*–*V* sweeps of the VLM, demonstrating a stable switching behavior with a resistance ratio *R*_HRS_/*R*_LRS_ ≈ 20. Note that the *R*_HRS_/*R*_LRS_ is estimated for the median *I*–*V* data, represented by the red solid line in Figure 1g. Compared to other laser-fabricated graphene-based memristors reported in the literature, the VLM exhibits a superior resistance-switching ratio with a comparable *V*_switch_ and the same maximum DC endurance (as shown later). Table 1 compares the performance of the VLM with some previously reported laser-fabricated graphene-based memristors. It should be highlighted that the VLM demonstrates volatile dynamic switching, whereas those reported in the literature in Table 1 are nonvolatile memristors.

**Table 1:** Table Comparing the Parameter of the VLM with Some of the Laser-Fabricated Graphene-based Memristors in the Literature

device	*V*_switch_	*R*_HRS_/*R*_LRS_	max. DC endurance	reference
Ag/HfO_*x*_/laser-scribed graphene	≈±1.6	10	100	(23)
Ag/laser fabricated GO/Ag	≈±2.5	6	100	(36)
Cu/LIG/Cu	±2	1.5	100	(35)
Cu/LIG-MnO_2_/Cu	±4	1.4	100	(35)
Al/LIG/Al	±3	20	100	this work

### Memristive Mechanism

**Figure 2:**
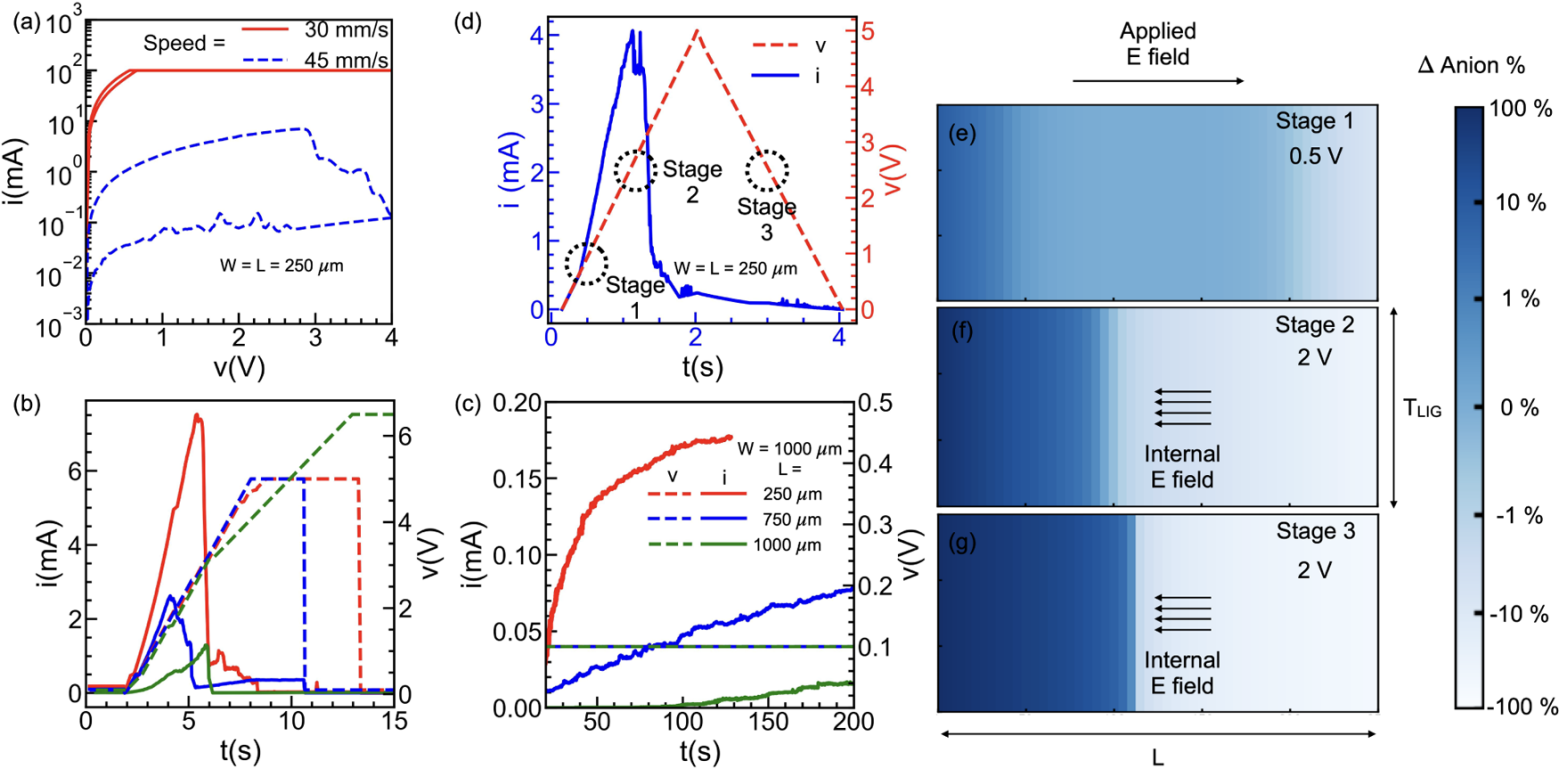
(a) Measured *I*–*V* characteristic of the LIG for two different laser engraving speeds: fast (45 mm/s; blue dashed line) shows resistive switching, and slower (30 mm/s; red line) does not show resistive switching but a much higher conductivity (current is limited to 100 mA to avoid structural damages). Switching characteristics of the VLMs as a function of time where (b) shows the switching from LRS to HRS and (c) showing the recovery of the VLMs toward LRS when voltage is kept constant at 0.1 V. (d) Measured current (blue line) and voltage (red dashed line) of the LIG sample as a function of time with 3 differentiated stages and the corresponding distribution of the ions, extracted from device simulations in (e) stage 1, (f) stage 2, and (g) stage 3. Here *L* is the length, and *T*_LIG_ is the thickness of the VLM.

Unlike high-quality CVD or exfoliated graphene, LIG does not result in a monolayer crystalline sp^2^ lattice of carbon atoms. The fabricated LIG contains structural defects and residual ions of oxygen or oxygen-containing species.^30,45^ The narrowing of the G peak and the reduction in the intensity of the D peak observed in the LIG Raman spectra (Figure 1d) suggest a reduction of both the structural defect and the oxygen content with increasing laser power. Therefore, to assess the role of the intrinsic defects in the observed resistive switching, *I*–*V* measurements were performed for VLM samples fabricated with different engraving speeds in Figure 2a. The slower one (30 mm/s) results in high conductivity and a lack of resistive switching, while the VLM prepared with a higher engraving speed (45 mm/s) shows much lower conductivity and resistive switching. This result supports the crucial role of residual defects and ions in generating the resistance-switching phenomena.

As seen earlier, the VLM exhibits a switch from LRS to HRS, indicating a nonfilamentary mode of operation.^12,46^ The VLM internal dynamics oppose the current flow, leading to a switch from high conductivity to low conductivity. Such clockwise/clockwise direction of switching for both voltage polarities could result from the presence of traps as well as ions modifying the internal electric field of the device.^46−48^ To distinguish between the role of traps and the ions in generating resistive switching, the dependence of the recovery time on the length of the VLM is analyzed. Figure 2b shows the voltage pulses applied to VLMs of increasing lengths (*L* = 250, 750, and 1000 μm). During the rising voltage sweep, the VLMs are in LRS and a high current flows through the VLM, before switching to HRS. After this switching, the voltage level is reduced and maintained at a low constant value of 0.1 V to monitor the current flowing through the VLMs without affecting the internal mechanism. Hence, Figure 2c depicts the current transient evolution for this constant voltage value of 0.1 V after the switching, proving the direct correlation between the recovery time (*t*_rec_) and the VLM length (*L*). This observed dependency corroborates the role of drift and diffusion of ions in generating resistive switching and rules out the possibility that interface or bulk traps cause memristive switching. Additionally, Figure 2b suggests that *t*_rec_ can be tuned by adjusting the VLM length, which may be conveniently reduced by scaling down the devices.

From the above observations, it is likely that the memristive effect is caused by a built-in electric field,^46^ generated due to the redistribution of residual defects or ions when an external bias is applied. To understand and visualize the ion dynamics during the switching process, additional device simulations are performed. An in-house developed numerical tool is employed that solves the electrostatics and time-dependent electronic and ionic transport self-consistently. The system of equations includes the Poisson equation and the time-dependent continuity equation for electrons and ions in the device. The details of the simulator can be found in the Supporting Information.

In the simulations, the negative ions (anions) are considered mobile, while the positive ions (cations) are stationary. To optimize computational time and due to limited information on material parameters, they were adjusted to align with the normalized trend of the experimentally obtained *I*–*V* (refer to Supporting Information). While not matching the experimental *I*–*V* shape exactly, the simulated *I*–*V* was able to replicate the observed clockwise switching of the resistance state. It is worth noting that reversing the polarity of the mobile species also results in the same switching trend. Based on the internal dynamics, the switching mechanism can be understood as follows. The experimentally measured transient current under the application of a triangular voltage sweep is shown in Figure 2d, while the corresponding anion distributions obtained from the device simulations at each applied bias are shown in Figure 2e–g, respectively. Note that Figure 2e–g shows the anion difference density with respect to their initial concentration at zero applied bias. During the rising voltage sweep (stage 1), the VLM is in an LRS and a high current flows through the LIG sample; however, simultaneously, mobile charged ions drift in the opposite direction of the applied external field. This results in a redistribution of the ions and the appearance of a built-in electric field inside the VLM, which opposes the external electric field (stage 2). Therefore, the net electric field perceived by the electrons diminishes and the VLM switches to an HRS. As a consequence, the current flowing through the VLM decreases, corresponding to stage 2 in Figure 2d, where the current is reduced by a factor of 10. When the external bias is diminished, during the falling voltage ramp (stage 3), the external electric field drops, and the ions start diffusing back, as can be seen from the shifting of the Δ anion peak in Figure 2g. However, since the ion diffusion is slow, the internal field drop is not instantaneous; a substantial nonuniform anion density persists, causing the electrons to keep moving under a reduced net electric field. Thus, the *I*–*V* characteristics feature a hysteretic behavior. On removal of the bias, ions eventually diffuse back to their initial positions after a recovery time and the VLM returns to the original LRS.

**Figure 3:**
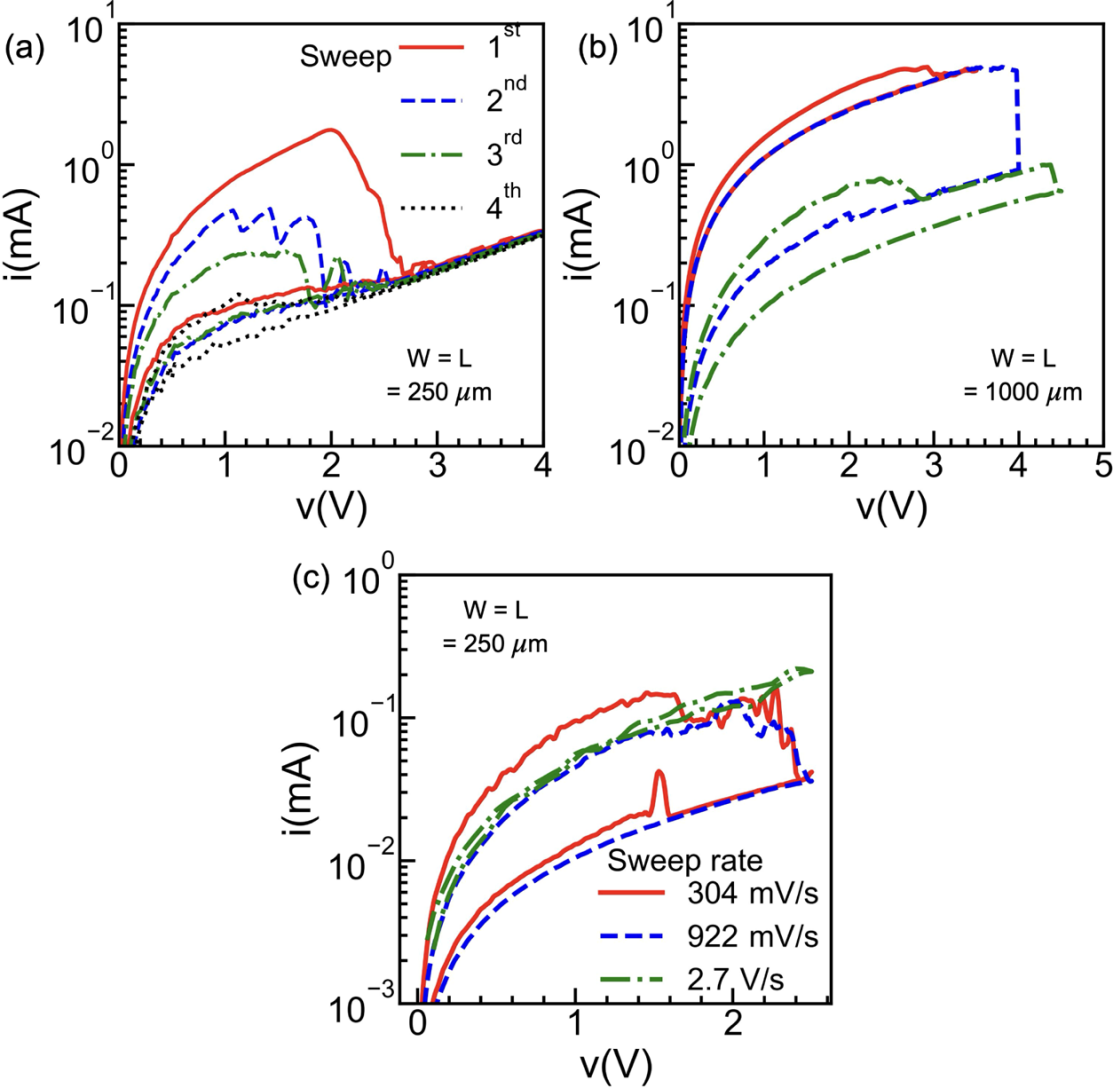
Repeated *I*–*V* measurement of the VLM with (a) rest time between subsequent sweeps smaller than the recovery time for the ions thereby reducing *R*_LRS_ in each cycle, (b) increasing maximum applied voltage (red: 3.5 V, blue: 4 V and green: 4.5 V) and hence maximum applied external electric field thereby increasing *R*_HRS_ in each cycle and (c) different voltage scan rates (red: 304 mV/s, blue: 922 mV/s and green: 2.7 V/s).

Different experiments were carried out to further support the aforementioned mechanism. Accordingly, the *R*_LRS_ value should get reinstated if enough time is provided for the ions to diffuse back to their original state, i.e., the recovery time. Then, if repeated *I*–*V* sweeps are carried out at time intervals shorter than the recovery time, a reduction of *R*_LRS_ will result after each sweep (in correspondence to the ions not recovering the equilibrium state). This is observed in Figure 3a: here, the off time (time interval between each voltage sweep) is kept to be less than the recovery time. Similarly, the change in the effective electric field and therefore the *R*_HRS_ value depend on the ions drift during the rising voltage sweep. In Figure 3b, repeated voltage sweeps with peak values of 3.5 4, and 4.5 V, applied with no off time, show that, if the peak voltage applied to the VLM is properly modulated, different values of the *R*_HRS_ can be achieved, also in line with the above mechanism. Additionally, the modulation of the *I*–*V* characteristics with varying voltage scan rates is observed in Figure 3c (additional measurements in Figure S3), which further corroborates the mechanism. As the voltage scan rate increases, ions are unable to follow the applied signal, leading to a reduced switching ratio. Eventually, the hysteresis disappears at sufficiently high scan rates.

### Statistical Measurements

**Figure 4:**
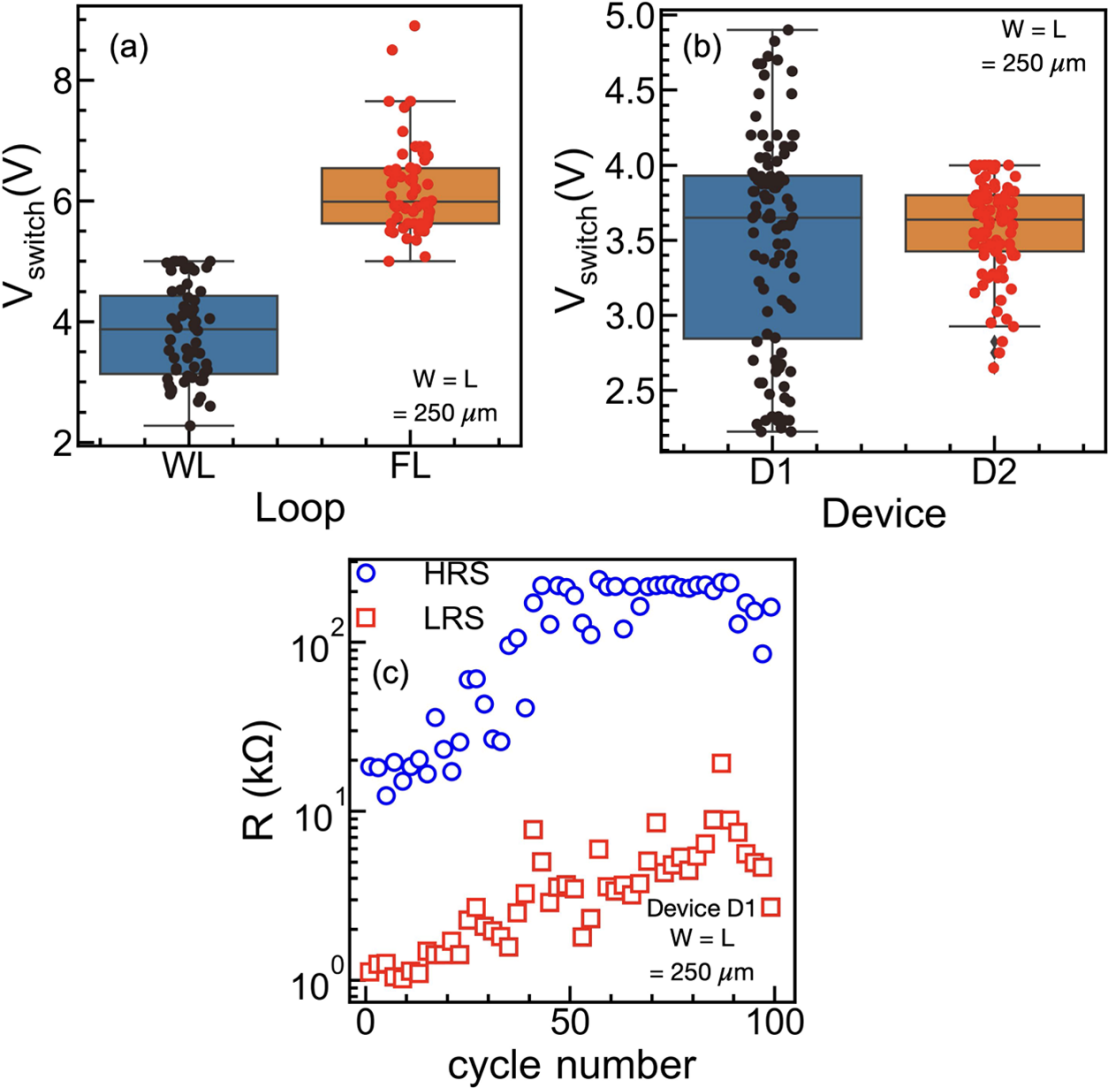
(a) Device-to-device variability of the VLMs for the forming loop (FL) and working loop (WL). Each point corresponds to a different fresh device. (b) Cycle-to-cycle variability of two different VLM devices for multiple consecutive *I*–*V* sweeps. (c) Endurance characteristics of the VLM showing distinguishable switching for 100 cycles.

Multiple *I*–*V* cycles were measured to evaluate the statistical variation of the fabricated VLMs. Figure 4a shows the device-to-device *V*_switch_ variation for the first forming loop (FL) and the working loop (WL). Each point in Figure 4a corresponds to measurements on a different fresh device with the boxplot enclosing *V*_switch_. Figure 4b shows the cycle-to-cycle variability of *V*_switch_ in the WL for two different VLM devices after multiple consecutive *I*–*V* sweeps. Although the variability is reasonable in device D2, device D1 shows a higher statistical spread of the *V*_switch_.

Further, the endurance is tested by extracting the resistance value from the full *I*–*V* sweep measurements performed on device D1, at a read voltage of 0.5 V. *R*_HRS_ (blue circles) and *R*_LRS_ (red squares) for 100 consecutive cycles are displayed in Figure 4c. Although the resistance values tend to increase with the cycling process, the VLM shows distinguishable resistance states, with a resistance-switching ratio of ≈20 over the 100 cycles. The full *I*–*V* curve for each sweep of the endurance test is provided in Figure S4. Interestingly, along with the resistance values, *V*_switch_ also changes from cycle to cycle, resulting into the high variability of D1 observed in Figure 4b.

### Circuit Design Application

**Figure 5:**
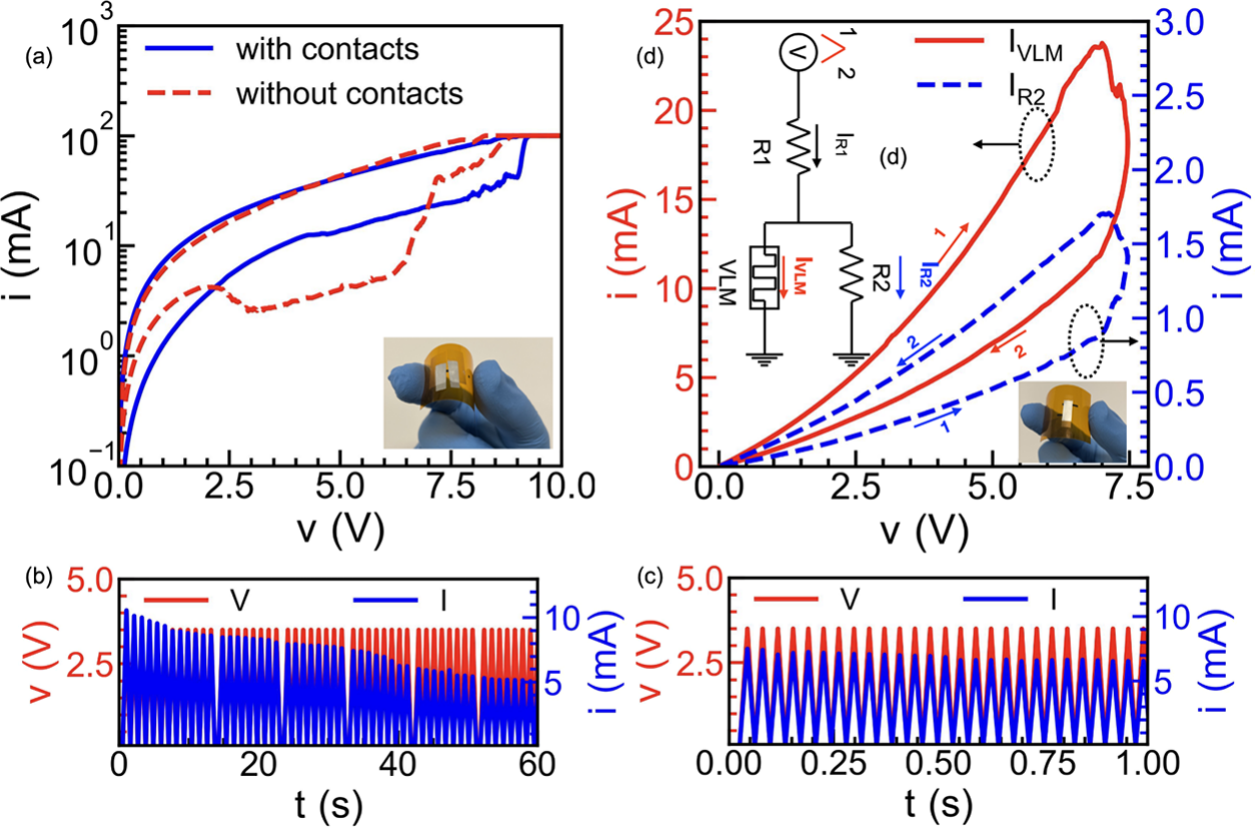
(a) Demonstration of the unaltered *I*–*V* characteristics after the use of aluminum sheet as contacts. Inset shows the fabricated device. Results of the pulse voltage stress measurements showing the experimental demonstration of PPD for a pulse period of (b) 1100 ms and (c) 38 ms, respectively. (d) Resistor network (inset) employed to obtain the *I*–*V* characteristics that shows the possibility of obtaining both, clockwise and anticlockwise hysteresis.

Aiming at testing the applicability of the VLMs as components of different circuits, they were connected by aluminum sheets. Figure 5a depicts the *I*–*V* characteristics of a VLM device with and without contacts, showing that the device retains its resistance-switching behavior.

The dynamic response of the device is analyzed by applying a pulse voltage stress (PVS), consisting of a series of consecutive triangular pulses with different periods as trigger input. Figure 5b shows the VLM current response when it is exposed to a PVS of amplitude 3.5 V and a period of 1100 ms. The output current through the VLM presents a pulse-dependent gradual change, suitable to emulate the relevant paired-pulse depression (PPD) featured by biological synapses.^10,49^ After each set of 12 (later 8) paired pulse trains, a 550 ms pause is set (equivalent to skipping half a pulse) to evidence that the VLM retains its state and resumes from the same maximum current value. The VLM can thus efficiently emulate the dynamics and characteristic time scale of the STP behavior of biological synapses, where the strength changes temporarily on a time scale ranging from milliseconds to minutes.^15^ Moreover, as shown in Figure 3b, the recovery time depends on the length of the device, which, unlike the filament-based memristors, provides a direct design knob to achieve a higher control of the time scales as both faster and slower recovery times can be achieved. In Figure 5b, the current changes from 10 mA at *t* = 0 s to 8 mA at the end of the first set of 12 pulses and eventually reduces to 5 mA after a total of 52 pulses. Figure 5c shows the VLM response for a significantly increased pulse rate where the period of the trigger pulses is 38 ms. The output current of the VLM still shows a pulse-dependent gradual change (from 7.5 mA at *t* = 0 s to 6.5 mA after 26 pulses). Both, Figure 5b,c further confirm the memristive mechanism of the VLM, providing additional support to the observations depicted in Figure 2f,2g.

Further, the time scale and the (pulse-dependent) state change of the VLM are highly suitable for the implementation of the LIF neuron,^38^ however, it naturally switches from LRS to HRS, i.e., in the clockwise direction. This is the opposite to the usual behavior of memristors used to implement the LIF neuron, which have anticlockwise switching. The anticlockwise switching in the VLM can be obtained with an all-LIG-based resistive network schematized in the inset of Figure 5d, which consists of two fixed-value resistors (R1 and R2) in addition to the switchable VLM. Notably, both the VLM and the resistors are fabricated from LIG, making use of the same procedure described in Results and Discussion section. The laser power and velocity, together with the length of the engraved regions, are adjusted to achieve either the desired value of the fixed resistor or the VLM, using aluminum sheets to interconnect them (see inset of Figure 5d). This implementation further exemplifies the remarkable flexibility and control of the LIG fabrication process. If a high current flows through the VLM (*I*_VLM_) in the LRS, then, the current through the parallel resistor R2 (*I*_R2_) remains low, while, when the VLM switches to a HRS and *I*_VLM_ has a lower value, *I*_R2_ increases to a higher one. The values of R1 and R2 were carefully selected such that the total current in the circuit (**I**_R1_ = *I*_VLM_ + *I*_R2_) remains nearly unaltered during the switching. Figure 5d shows the current through R2, with the sought anticlockwise hysteresis. This topology can also be combined with a nonvolatile element to implement long-term plasticity and the other desired learning rules.^10^

## Conclusion

A cost-effective patterning and transfer-free laser-induced graphene (LIG) process was used to implement memristive devices directly on a commercial flexible polyimide substrate. For the first time, volatile resistance switching was demonstrated in a LIG-only memristor without the incorporation of additional materials. The fabricated prototypes (VLM) were electrically characterized, generating reliable resistance-switching characteristics. Compared to the previous laser-fabricated graphene-based memristors reported in the literature, the VLM featured an improved resistance switching ratio of 20 at a comparable switching voltage of ±3 V and an *I*_max_/*I*_off_ ratio around 10^3^ with distinguishable resistance switching for 100 cycles. Although promising results were obtained, the VLM exhibited reasonable variability, which suggests that further optimization of the contacts and fabrication process is required. Based on the clockwise hysteresis shown by the VLMs, corroborated with numerical device simulations, the potential mechanism giving rise to the device switching was attributed to the built-in electric field originated by the redistribution of defects and ions. The measured *I*–*V* characteristics and the dependence of the resistance values on the recovery time, which were found to be a function of the device length, agreed well with the proposed mechanism. The dynamic response of the VLM showing the input-pulse-dependent state change was shown to emulate short-term plasticity schemes such as paired-pulse depression (PPD). Finally, the possibility to achieve greater control over the material properties by directly tuning the laser power was demonstrated through the implementation of an all-LIG circuit to reverse the switching direction. A simple circuit designed to obtain a standard anticlockwise hysteresis shows the potential to implement different learning rules and components akin to biological synapses.

## Experimental Section

### Device Fabrication

Commercial flexible polyimide (Kapton sheets, 150 μm thick DuPont 300HN) were employed as substrate for the device fabrication. A high-precision diode-pumped laser with a 532 nm wavelength (Coherent Powerline E12 SHG) was used for scribing the polyimide film. The resolution of the galvanometric positioning system limits the minimum dimensions of the fabricated devices to 250 μm.

### Structural Characterization

The Raman spectroscopy was carried out using a JASCO NRS-5100 micro-Raman Dispersive Spectrometer, with an excitation source of λ = 532 nm (Elforlight G4-30; Nd:YAG) and 30 mW power. The NX20 Atomic Force Microscope from Park Systems was used to capture the Atomic Force Microscopy (AFM) images of the laser-scribed region. The optical microscope installed in the electrical characterization setup was used to capture the closed-up image of the fabricated samples.

### Electrical Characterization

The electrical characterization was carried out using an EverBeing C-4 Probe Station connected to a Keithley 4200A-SCS parameter analyzer. Initially, the tungsten probes of the SMUs were directly employed to contact the material, as schematically shown in Figure 1a, whereas the use of aluminum as contacts is demonstrated later.

## Supporting Information Available

Further details on the numerical simulations and additional measurements (PDF)
