# Supplementary material for: A Flexible Laser-Induced Graphene Memristor with Volatile Switching for Neuromorphic Applications
Source: ACS Appl Mater Interfaces. 2024 Sep 6;16(37):49724–32. doi: 10.1021/acsami.4c07589 (PMC11420864; doi:10.1021/acsami.4c07589)
Supplement: Supplementary file 1 — am4c07589_si_001.pdf [file am4c07589_si_001.pdf]

Supporting Information

A flexible laser induced graphene memristor with volatile switching for neuromorphic applications

Mohit D. Ganeriwala,* Roberto Motos Espada, Enrique G. Marin, Juan Cuesta-Lopez, Mikel Garcia-Palomo, Noel Rodríguez, Francisco G. Ruiz, and Andres Godoy*

Electronics Department, Campus Fuentenueva S/N, University of Granada, Granada 18071, Spain.

E-mail: mohit@ugr.es; agodoy@ugr.es

Polyimide chemical structure

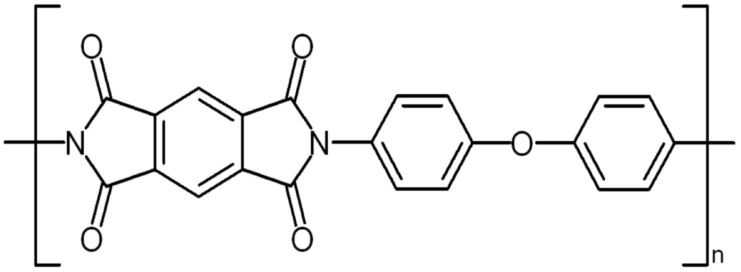

[poly(4,4′-oxydiphenylene-pyromellitimide)]

Figure S1: Chemical structure of the polyimide (Kapton® sheets, DuPont™ 300HN), showing chain of carbon with C-O, C=O and C-N bonds, which could be broken by localized heating.

Device simulations

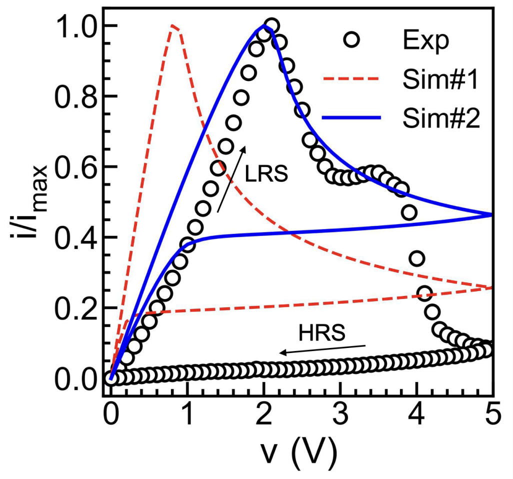

Parameters	Sim #1	Sim #2
L	250 nm	
T_{LIG}	150 nm	
W	250 nm	
μ_n	20 cm²/Vs	
ϵ_{LIG}	7.6	
N_D	0	
z	[+1,-1]	[+1,-1]
c_i	[2,1]x10^{20} cm^{-3}	[2,1] x 10^{18} cm^{-3}
μ_i	[0,5] x 10^{-11} cm²/Vs	[0,1] x 10^{-10} cm²/Vs

Figure S2: Experimental (symbols) and simulated (solid lines) I-V characteristics of the volatile LIG memristors for two different materials parameters denoted by Sim#1 and Sim#2. Table gathering the simulation parameters.

Device simulations have been carried out employing an *in-house* numerical tool that solves self-consistently the electrostatics and time-dependent electronic and ionic transport under a semi-classical scheme. The system of equations comprises the Poisson equation (eq. 1), the time-dependent continuity equation for electrons and ions in the device,[1] following a drift-diffusion scheme after applying the Scharffetter-Gümmel method (eq. 3).[2]

$$\nabla(\varepsilon \nabla V) = -\left(N_{\mathrm{D}} - n + \sum_{\mathrm{i}} z_{\mathrm{i}} c_{\mathrm{i}} \right) \tag{1}$$

$$\vec{\nabla} \cdot \vec{J_n} = \vec{\nabla} \cdot \left[q\mu_n n \vec{\nabla} E_{\mathrm{F,n}} \right] = +q\frac{\partial n}{\partial t} \tag{2}$$

$$\vec{\nabla} \cdot \vec{J_i} = \vec{\nabla} \cdot \left[-z_i q D_i e^{-s_i \Phi} \vec{\nabla} \left(c_i e^{s_i \Phi} \right) \right] = -z_i q \frac{\partial c_i}{\partial t} \tag{3}$$

where V is the electrostatic potential, ε is the dielectric constant, $\vec{J}$ is the current density, q is the elementary charge unit, μ_n is the electron mobility, n is the electron density, E_F is the pseudo-Fermi energy level, t is the time, z_i is the ion valence with $s_i = |z_i|/z_i$ the ion

valence sign, c_i is the ion concentration, D_i is the ion diffusion coefficient, which follows the Einstein relationship ($D_i = \mu_i \frac{k_\mathrm{B}T}{q}$), k_B is the Boltzmann constant, T is the temperature, $\Phi = \frac{qV}{k_\mathrm{B}T}$ is the normalized potential, and the sub-indexes n and i denote electrons and each ion species, respectively.

The system of equations was solved using a finite difference scheme for a two terminal device with the dimensions shown in Fig. 1g of the manuscript. The electron density is calculated using the Fermi-Dirac statistics and considering the graphene density of states. At the initial instant it is assumed that ions and electrons are uniformly distributed across the device, making every single point in the device charge neutral at zeroth time. The boundary conditions are applied such that ions are confined inside the device. More details on the use of drift-diffusion numerical simulations for memristive devices could be found in.[3] The fabricated LIG contains structural defects and residual ions of oxygen or oxygen-containing species.[4,5] To align with the same in the simulations, the negative ions (anions) are considered mobile while the positive ions (cations) are stationary. However, it is worth noting that reversing the polarity of the mobile species also results into the same switching trend. A considerably smaller time step is required to accurately converge the simulations, which increases the computational workload. Moreover, there is limited information available on the material parameters of the LIG, such as the type and mobility of the ions, which need to be adjusted to match the experimental characteristics. Thus, this simulation study focuses on the explanation of the observed resistive switching, which shows a transition from a low resistance state to a high resistance state, i.e., a clockwise hysteresis in the I-V characteristics. A scaled device is simulated using the geometrical parameters shown in Fig. S3. The obtained characteristics for two different choices of material parameters are compared with the experimentally measured I-V (symbols). Note that, as explained earlier, the trend was captured by comparing normalized current values. We observed that by adjusting the material parameters, different characteristics can be obtained. Although not an exact match with the experiments, the simulated I-V was able to replicate the observed clockwise switching of

the resistance state, providing additional theoretical support to our analysis of the intrinsic relationship between material features, device dimensions, and switching behaviour.

Measurments with varying voltage scan rate

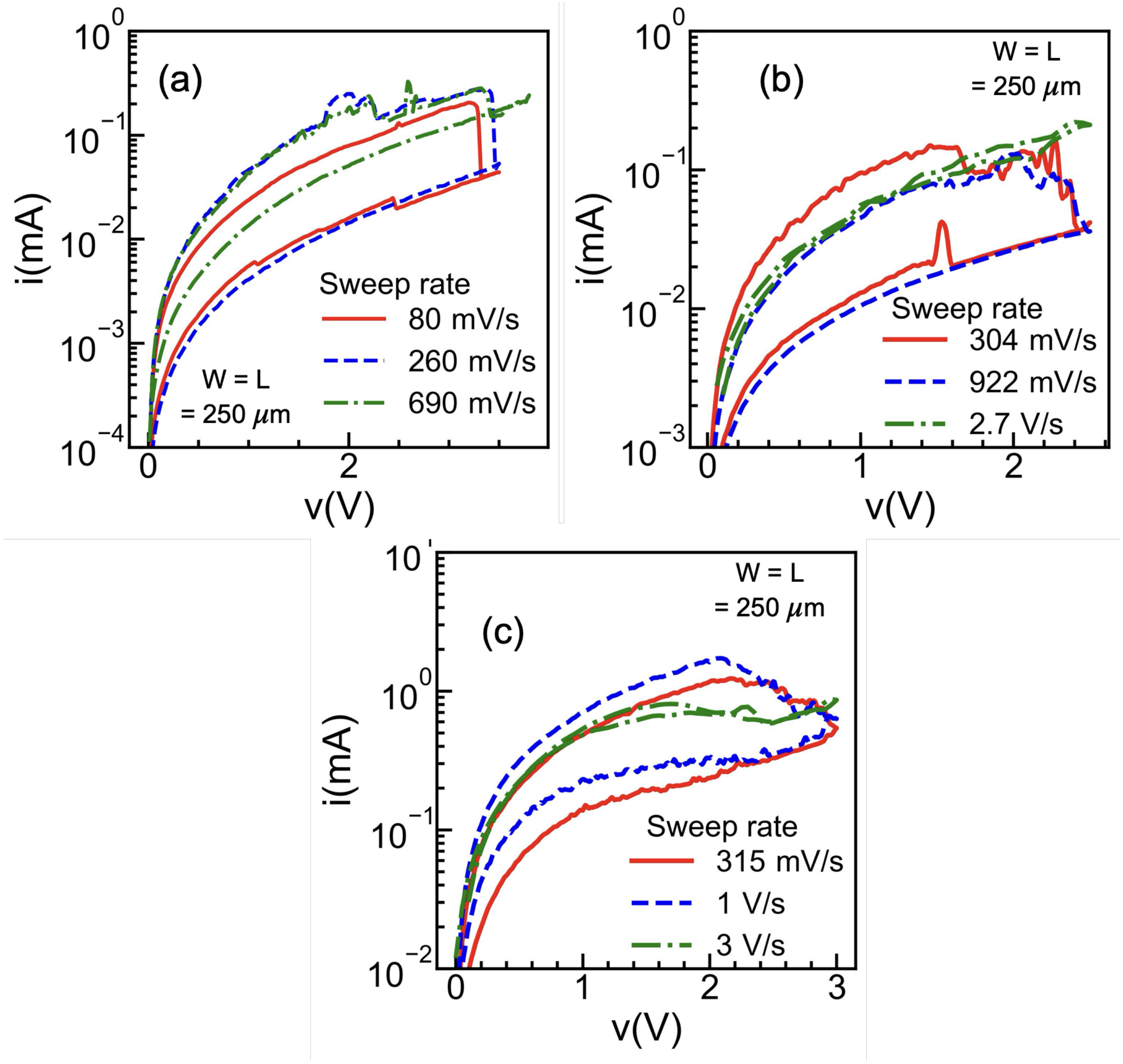

Figure S3: I-V loops of VLM measured at different voltage scan rate. The device behavior changes with the scan rate, exhibiting a change in the switching window with increasing scan rate. With an increasing voltage scan rate, the ions can no longer follow the applied signal, resulting in the eventual disappearance of hysteresis at a sufficiently high scan rate.

Endurance Test

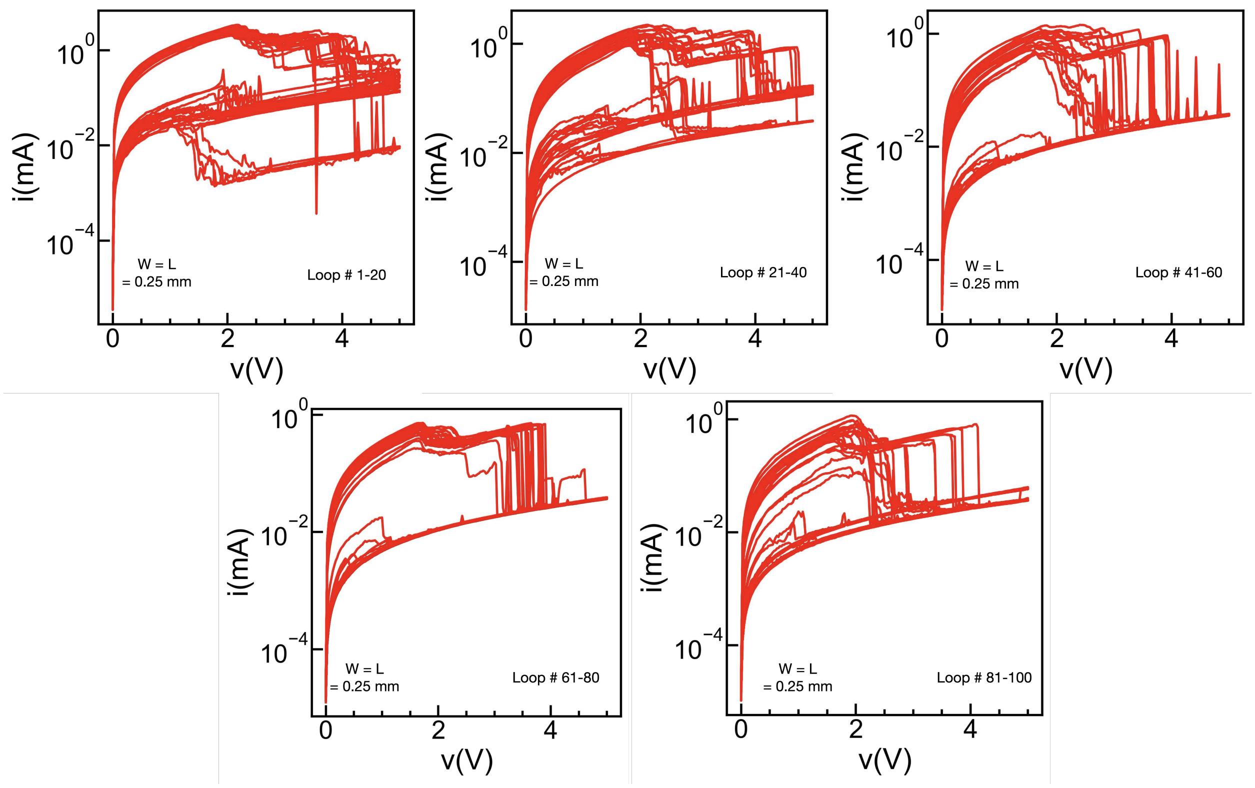

Figure S4: 100 I-V loops employed for the endurance test divided in five figures of 20 cycles each for clarity.

References

(1) Sze, S.; Li, Y.; NG, K. *Physics of semiconductor devices*; Wiley, 2012.

(2) Scharfetter, D.; Gummel, H. Large-signal analysis of a silicon Read diode oscillator. *IEEE Transactions on Electron Devices* **1969**, *16*, 64–77.

(3) Spetzler, B.; Abdel, D.; Schwierz, F.; Ziegler, M.; Farrell, P. The Role of Vacancy Dynamics in Two-Dimensional Memristive Devices. *Advanced Electronic Materials* **2024**, *10*, 2300635.

(4) Le, T.-S. D.; Phan, H.-P.; Kwon, S.; Park, S.; Jung, Y.; Min, J.; Chun, B. J.; Yoon, H.; Ko, S. H.; Kim, S.-W.; others Recent Advances in Laser-Induced Graphene: Mechanism, Fabrication, Properties, and Applications in Flexible Electronics. *Advanced Functional Materials* **2022**, *32*, 2205158.

(5) Vashisth, A.; Kowalik, M.; Gerringer, J. C.; Ashraf, C.; van Duin, A. C. T.; Green, M. J. ReaxFF Simulations of Laser-Induced Graphene (LIG) Formation for Multifunctional Polymer Nanocomposites. *ACS Applied Nano Materials* **2020**, *3*, 1881–1890.
